# Dynamic Modeling and Frequency Characteristic Analysis of a Novel 3-PSS Flexible Parallel Micro-Manipulator

**DOI:** 10.3390/mi12060678

**Published:** 2021-06-10

**Authors:** Jun Ren, Qiuyu Cao

**Affiliations:** School of Mechanical Engineering, Hubei University of Technology, Wuhan 430068, China; caoqiuyu20210517@163.com

**Keywords:** 3-PSS, flexible spherical hinge, flexible parallel micro-manipulator, dynamics

## Abstract

Dynamic modeling and frequency characteristic analysis of a novel 3-PSS (three-prismatic-spherical-spherical) flexible parallel micro-manipulator with three translational DOF in space were investigated in this paper. Firstly, the kinematics analysis was developed based on the pseudo-rigid body model. The Jacobian matrix and the relationship between the micro angular deformation of the flexible spherical hinge and the end pose of mobile platform were respectively obtained by employing vector closed-loop method and coordinate transformation method. Then, taking into account the elastic strain energy of the flexible spherical hinge, dynamic model of this mechanism was established via Lagrange equations, and the expression of natural frequency was further derived. Combined with a set of given parameters, natural frequencies of the system were calculated by using MATLAB software. For the comparison purpose, a simulated modal analysis of the mechanism with the same parameters was also performed by employing finite element ANSYS software. Results from numerical calculation and finite element simulation indicated that maximum error of their natural frequencies was 2.71%, which verified the correctness of the theoretical dynamic model. Finally, variations of natural frequencies with changes of the basic parameters were analyzed. Analysis results show that natural frequencies increase with the increase of the bending stiffness *k_bm_* of flexible spherical hinge and the difference in radius *E_r_* between static platform and mobile platform, while decrease with the increase of the length *l* of the link rod and the masses of the main components of mechanism. Besides, it can be further drawn from these obtained results that the natural frequencies increase with the increase of the angle *θ_l_* between the link rod and the *z* axis of reference coordinate system. Considering that the increase of the stiffness *k_bm_* and the angle *θ_l_* will reduce the scope of working space, it is recommended in designing the structure to choose a set of larger stiffness *k_bm_* and angle *θ_l_* as much as possible under the premise of satisfying the working space.

## 1. Introduction

The flexible parallel micro-manipulator combines a series of advantages of parallel mechanism and flexible mechanism including stable structure, high precision, small error, no backlash, and zero friction. Thus, it is widely used in fields such as precise positioning, microelectronics, nano-manipulation, micro-manufacturing, micro-operation of optoelectronic components and biomedical engineering [1,2,3,4]. Researches on flexible parallel micro-manipulator mainly refer to structural design, kinematic analysis, static stiffness analysis and dynamic analysis [5,6,7,8,9,10]. Among them, dynamic analysis is the basic condition of mechanism optimization design and high frequency control [11,12], and it is also one of the key and difficult points in the research of flexible parallel micro-manipulators.

Before performing kinematics or dynamics analysis on a flexible mechanism, it is usually necessary to convert it to an equivalent rigid body model. In the early, Howell [13] proposed the pseudo-rigid-body method that equates the flexible mechanism to an equivalent rigid body mechanism for the design and analysis of flexible mechanisms. After that, some researchers integrated this idea into the kinematics analysis of flexible parallel micro-manipulation mechanism by equating the flexible hinges to kinematic pairs with constant stiffness [14,15]. Some other scholars employed similar equivalent processing methods to investigate the dynamics of flexible parallel mechanisms with different configurations. Xu [16] designed a parallel XY micromanipulator for two-dimensional (2-D) micromanipulation applications, and established the mathematical models predicting kinematics, statics, and dynamics of the XY stage based on the pseudo-rigid body simplification approach. Tian [17] presented a 3-RRR flexible parallel micromanipulator with three-degree-of-freedom, and established its inverse dynamic equation using Lagrangian equation by equating the rotating hinges to rotating pairs with constant stiffness. Based on pseudo-rigid body model, Jia established dynamic model of 3-RPRR flexible parallel mechanism by Lagrangian method [18] and virtual work principle method [19], respectively. Qi [20] proposed a 2-DOF compound compliant parallel guiding mechanism. The elastic deformation potential energy of the flexible hinge was firstly solved based on the pseudo-rigid body model, and then the dynamic model of the mechanism was established by using Lagrangian equations. Li [21] proposed a 3-PRC compliant parallel micromanipulator with three degrees of freedom. The kinematics model was firstly derived using vector method, and dynamic equation was then established by the Lagrangian method. Most of the above-mentioned flexible parallel mechanisms use planar flexible hinges with single degree of freedom as the flexible component, while space flexible spherical hinge with three degrees of freedom is rarely involved. A key part of the dynamic modeling of the flexible parallel mechanism is to solve the elastic deformation potential energy expression of the flexible component. Compared with flexible hinge that only moves in a plane, the process of solving the elastic potential energy expression of the flexible spherical hinge with spatial motion is more complicated.

In this paper, a novel type of flexible parallel micromanipulator with flexible spherical hinges is proposed. Idea of designing this mechanism originates from replacing the ordinary spherical hinges in the 3-PSS parallel mechanism with flexible spherical hinges. Focus of this paper is investigating dynamic modeling and frequency analysis of this 3-PSS flexible parallel micromanipulator. Firstly, based on the pseudo rigid body model, the Jacobian matrix is obtained by using vector closed-loop method, and relationship between angular displacement of the flexible spherical hinge and end pose of end-effector (mobile platform) is gained by using coordinate transformation method. Then, incorporating elastic strain energy of the flexible spherical hinges, the dynamic equation of the mechanism is formulated using the Lagrangian equation, by which expression of the natural frequency of this mechanism is further derived. Finally, the influence of variations of the stiffness of the flexible spherical hinge, the mass of the main component and main structural parameters on the natural frequencies is analyzed.

## 2. Structure of Micro-Manipulator

**Figure 1:**
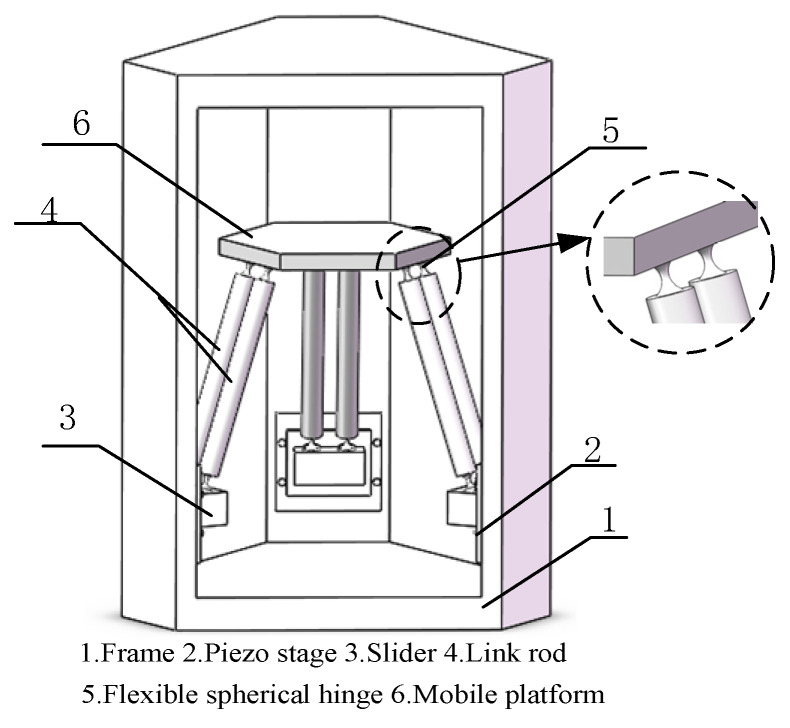
The structure of 3-PSS flexible parallel micro-manipulator.

The most commonly used design method for the structure of flexible parallel micro-manipulators is to replace the hinges in the traditional parallel mechanism with flexible hinges [22,23]. In this paper, the flexible parallel micro-manipulator is designed by replacing the ordinary spherical hinges in the 3-PSS type delta parallel mechanism with flexible spherical hinges. As is shown in Figure 1, the micro-manipulator is composed of a mobile platform, a fixed frame, the sliders, and three identical limbs distributed symmetrically at 120°. Each limb has two parallel link rods *B_ij_P_ij_* (*i* = 1, 2 and 3; *j* = 1, 2), which are respectively connected to the slider and the mobile platform through flexible spherical hinges. Three sliders are respectively fixedly connected with the moving stages of the piezo stages, and move with them. The fixed frame of the piezo stage is connected with the vertical rail on frame of the mechanism.

## 3. Inverse Kinematic Analysis

**Figure 2:**
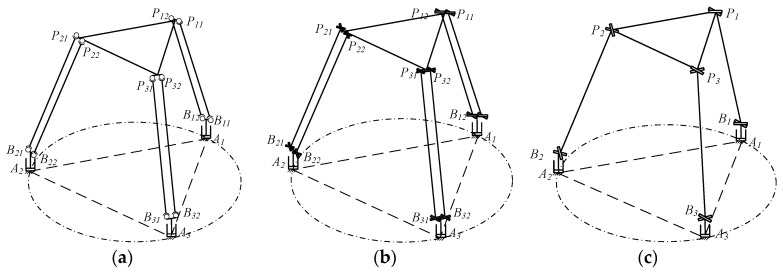
The equivalent process of the motion diagram. (**a**) Motion diagram. (**b**) PRB model. (**c**) Simplified PRB model.

Kinematic analysis is the premise and foundation of dynamic modeling. When the motion regularity of end effector (mobile platform) is known, the motion characteristics of other components of mechanism can be solved, which is so-called “inverse kinematics analysis”. Motion diagram of this flexible parallel micro-manipulator is given in Figure 2a. According to screw theory [24], the torsional degree of freedom of the flexible spherical hinges in this flexible parallel micro-manipulator is restricted, which results in each flexible spherical hinge having only two rotational degrees of freedom. Each flexible hinge can thus be simplified into two orthogonal ideal revolute joints with constant bending stiffness, the axes of which are all perpendicular to the central axis of the link rod *B_ij_P_ij_*. Then, the pseudo rigid body (PRB) model of this micro-manipulator can be established, as is shown in Figure 2b. Considering that the motion characteristics of the two link rods *B_ij_P_ij_* of the same limb have identical motion, each limb can be regarded as one rigid link rod *B_i_P_i_* (hereinafter referred to as “equivalent rod *B_i_P_i_*”) for the sake of simplicity of the analysis, as can be seen in Figure 2c. Obviously, the length and motion characteristics of the equivalent rods *B_i_P_i_* are the same as the original link rod *B_ij_P_ij_*, and the mass is twice that of the original link rod *B_ij_P_ij_*. And the two flexible spherical hinges at each end of the original limb can be equivalent to one spherical hinge (referred to as “equivalent spherical hinge”), whose deformation is equal to that of the original flexible spherical hinge. The only difference is that the bending stiffness of the equivalent spherical hinge is equal to twice that of the original flexible spherical hinge. In order to facilitate the analysis, the following inverse kinematics analysis can be carried out based on the simplified pseudo-rigid body (PRB) model in Figure 2c.

**Figure 3:**
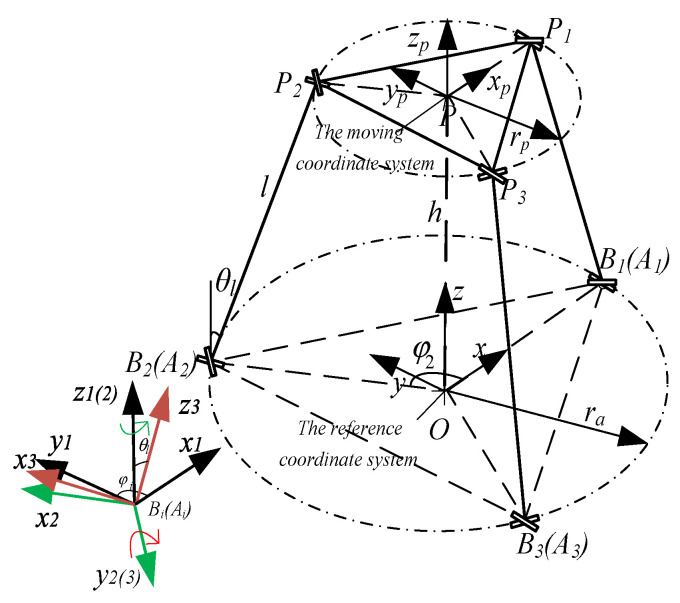
The coordinate system diagram of simplified PRB model.

In order to simplify the analysis, the equilateral triangle formed by the initial centroids *A_i_* (*i* =1, 2, 3) of the three sliders is defined as the static platform of the mechanism. The reference coordinate system *O*{*x*, *y*, *z*} is attached at the center point *O* of the static platform. The moving coordinate system *P*{*x_p_*, *y_p_*, *z_p_*} is established at the center point *P* of the mobile platform, whose *x*-axis is parallel to the *x*-axis of the reference coordinate system, and its *z*-axis coincides with the *z*-axis of the reference coordinate system. The radius of the static platform and the mobile platform are *r_a_* and *r_p_*, respectively. In the initial state, the vertical distance between the mobile platform and the static platform is *h*. The length of each link rod *B_i_P_i_* is *l*, where the point *B_i_* and the point *P_i_* respectively represent the center points of the flexible spherical hinges at two ends of the link rod. It should to be noted that the centroid of lower flexible spherical hinge coincides with the centroid of the slider, so *A_i_B_i_* represents the input displacement of the *i*-th slider. *OA_i_* is the position vector from the center point *O* of the static platform to the initial centroid point *A_i_* of the slider, and *φ_i_* (*φ*_1_ = 0, *φ_i_*_+ 1_ = *φ_i_* + 2/3π) is the angle between *OA_i_* and the *x*-axis of the reference system. The angle between the axis of link rod *B_i_P_i_* and the *z*-axis of the reference coordinate system is defined as *θ_l_*, as shown in Figure 3.

### 3.1. Jacobian Matrix

For this micro-manipulator, the mobile platform translates small displacement *x*, *y*, *z* in the reference coordinate system. So the transformation matrix of the moving coordinate system *P*{*x_p_*, *y_p_*, *z_p_*} relative to the reference coordinate system *O*{*x*, *y*, *z*} can be expressed as

(1) TPO=[100x010y001z+h0001]T

As shown in Figure 3, a vector-loop equation can be written for *i*-th limb of this flexible parallel micro-manipulator as follows.

(2) $$P_{i}P+PO+OA_{i}+A_{i}B_{i}=P_{i}B_{i}$$

And then the Equation (2) can be expanded into the following forms

(3) $${\left(x-E_{r}c\varphi_{i}\right)}^{2}+{\left(y-E_{r}s\varphi_{i}\right)}^{2}+{\left(z+h-b_{i}\right)}^{2}=l^{2}$$

with the notation of

$$E_{r}=r_{a}-r_{p}$$

where *sφ_i_* represents *sinφ_i_*, *cφ_i_* represents *cosφ_i_*, and the others are the same. The *b_i_* represents the input distance of *i*-th slider.

Taking the derivative of Equation (3) with respect to time, writing the equation three times, once for each *i* = 1, 2, and 3, yields three scalar equations which can be written in the matrix form as

(4) $$J_{1}\dot{B}=J_{2}\dot{P}$$

were $\dot{B}={\left[\begin{array}{ccc} {\dot{b}}_{1} & {\dot{b}}_{2} & {\dot{b}}_{3} \end{array}\right]}^{T}$ represents the speed of the slider, $\dot{P}={\left[\begin{array}{ccc} \dot{x} & \dot{y} & \dot{z} \end{array}\right]}^{T}$ represents the speed of the origin *P* of the mobile platform. And where

$$\begin{array}{c} J_{1}=diag(\begin{array}{ccc} z+h-b_{1} & z+h-b_{2} & z+h-b_{3} \end{array})\\ J_{2}=\left[\begin{array}{ccc} x-E_{r}c\varphi_{1} & y-E_{r}s\varphi_{1} & z+h-b_{1}\\ x-E_{r}c\varphi_{2} & y-E_{r}s\varphi_{2} & z+h-b_{2}\\ x-E_{r}c\varphi_{3} & y-E_{r}s\varphi_{3} & z+h-b_{3} \end{array}\right] \end{array}$$

The following velocity equations can be derived from Equation (4).

(5) $$\dot{B}=J_{1}^{-1}J_{2}\dot{P}=J\dot{P}$$

where ***J*** is the 3 × 3 Jacobian matrix of the 3-PSS flexible parallel micro-manipulator.

Considering that the unit of the input displacement *b_i_* and output displacement *x*, *y*, *z* of this micro-manipulator is micrometers, where the unit of *E_r_* and *h* is millimeters. Thus, the Jacobian matrix can be approximately expressed as

(6) $$J\approx \left[\begin{array}{ccc} -E_{r}c\varphi_{1}/h & -E_{r}s\varphi_{1}/h & 1\\ -E_{r}c\varphi_{2}/h & -E_{r}s\varphi_{2}/h & 1\\ -E_{r}c\varphi_{3}/h & -E_{r}s\varphi_{3}/h & 1 \end{array}\right]$$

### 3.2. The Relationship between the Micro Angular Deformation of the Flexible Spherical Hinge and the End Pose of Mobile Platform

According to Figure 2, analyzing the relationship between the angular displacement of the flexible spherical hinge and the end pose of the mobile platform can be transformed into analyzing the relationship between the angular displacement of equivalent spherical hinge and the end pose of the mobile platform.

As shown in Figure 3, for ease of calculation, the local coordinate system including $B_{i}^{1}${*x*_1_*, y*_1_*, z*_1_}, $B_{i}^{2}${*x*_2_, *y*_2_, *z*_2_} and $B_{i}^{3}${*x*_3_, *y*_3_, *z*_3_} are assigned to the center point *B_i_* of the flexible spherical hinges at the lower ends equivalent rod *B_i_P_i_*. The coordinate system $B_{i}^{1}$ {*x*_1_*, y*_1_*, z*_1_} is obtained by translating the reference coordinate system *O*{*x*, *y*, *z*} to point *B_i_*. The coordinate system $B_{i}^{2}${*x*_2_, *y*_2_, *z*_2_} is obtained through a series of coordinate transformations of the local coordinate system $B_{i}^{1}${*x*_1_*, y*_1_*, z*_1_}, and its *z*-axis direction is the same as the initial vector direction of the equivalent rod *B_i_P_i_*. In the initial state of the micro-manipulator, the coordinate system $B_{i}^{3}${*x*_3_, *y*_3_, *z*_3_} that changes with the motion of the flexible spherical hinge coincides with the coordinate system $B_{i}^{2}${*x*_2_, *y*_2_, *z*_2_}.

According to the established coordinate system, the coordinate transformation matrix of the coordinate system $B_{i}^{1}${*x*_1_*, y*_1_*, z*_1_} relative to reference coordinate system *O*{*x*, *y*, *z*} is

(7) $${}_{B_{i}^{1}}^{O}T=\left[\begin{array}{cccc} 1 & 0 & 0 & r_{a}c\varphi_{i}\\ 0 & 1 & 0 & r_{a}s\varphi_{i}\\ 0 & 0 & 1 & b_{i}\\ 0 & 0 & 0 & 1 \end{array}\right]$$

The matrix ${}_{B_{i}^{2}}^{B_{i}^{1}}T$ represents the homogeneous transformation matrix of the coordinate system $B_{i}^{2}${*x*_2_, *y*_2_, *z*_2_} respect to the coordinate system $B_{i}^{1}${*x*_1_*, y*_1_*, z*_1_}. It can be obtained by twice coordinate transformations of the coordinate system $B_{i}^{1}${*x*_1_*, y*_1_*, z*_1_}. The two coordinate transformations are the coordinate system $B_{i}^{1}${*x*_1_*, y*_1_*, z*_1_} first rotate around the *y*_2_ axis by -*θ_l_* angle, and then around the *z*_2_ axis by *φ_i_* angle. Therefore, the matrix ${}_{B_{i}^{2}}^{B_{i}^{1}}T$ can be expressed as

(8) $${}_{B_{i}^{2}}^{B_{i}^{1}}T=\left[\begin{array}{cccc} c\varphi_{i}c\theta_{l} & -s\varphi_{i} & -c\varphi_{i}s\theta_{l} & 0\\ s\varphi_{i}c\theta_{l} & c\varphi_{i} & -s\varphi_{i}s\theta_{l} & 0\\ s\theta_{l} & 0 & c\theta_{l} & 0\\ 0 & 0 & 0 & 1 \end{array}\right]$$

It is assumed that two angular displacements of the equivalent flexible spherical hinge at *B_i_* are *α_i_* and *β_i_*, respectively. Since the angular displacement of the flexible spherical hinge is small, *sα* ≈ *α*, *cα* ≈ 1, *sβ* ≈ *β*, *cβ* ≈ 1, and the infinitesimal terms are ignored. Thus, the coordinate transformation of coordinate system $B_{i}^{3}${*x*_3_, *y*_3_, *z*_3_} relative to coordinate system $B_{i}^{2}${*x*_2_, *y*_2_, *z*_2_} can be expressed as

(9) TBi3Bi2=[10βi001−αi0−βiαi100001]

In summary, the transformation matrix of coordinate system $B_{i}^{3}${*x*_3_, *y*_3_, *z*_3_} relative to reference coordinate system *O*{*x*, *y*, *z*} is

(10) Bi3OT=TBi1OTBi2Bi1TBi3Bi2

The coordinate of point *P_i_* in the local coordinate system $B_{i}^{3}${*x*_3_, *y*_3_, *z*_3_} is $P_{i}^{B_{3}}=\left(\begin{array}{ccc} 0 & 0 & l \end{array}\right)$. Thus, in the reference coordinate system *O*{*x*, *y*, *z*}, the coordinate of point *P_i_* can be expressed as

(11) [xpiypizpi1]=TBi3O[00l1]

The coordinate of point *P_i_* in the moving coordinate system *P*{*x_p_*, *y_p_*, *z_p_*} is $P_{i}^{P}=\left(\begin{array}{ccc} r_{p}c\varphi_{i} & r_{p}s\varphi_{i} & 0 \end{array}\right)$. Then, the coordinate of point *P_i_* in the reference coordinate system *O*{*x*, *y*, *z*} can also be expressed as

(12) [xpiypizpi1]=TPO[rpcφirpsφi01]

By making the corresponding terms at the right ends of Equations (11) and (12) equal, the angular displacement (α*_i_* and β*_i_*) of the flexible spherical hinge can be obtained.

Note that the flexible spherical hinge does not deform when the mobile platform moves along the *z*-axis direction, which can be seen from the Equation (6). Thus, relationship between angular displacement of flexible spherical hinge and end pose of mobile platform is

(13) $$\psi_{i}=J_{\psi i}P$$

where $\psi_{i}={\left[\begin{array}{ccc} \alpha_{i} & \beta_{i} & 0 \end{array}\right]}^{T}$, $J_{\psi i}$ is the 3×3 Jacobian matrix of angular displacement $\psi_{i}$ of flexible spherical hinge.

## 4. Dynamic Modeling

Lagrange’s equation of motion is adopted for the dynamics modeling of the 3-PSS flexible parallel micro-manipulator. Variable *q* is chosen as the generalized coordinates. Both the potential and kinetic energies of the micro-manipulator should be expressed in terms of the selected coordinates for obtaining the dynamic model. It is well known that all kinetic energy of the micro-manipulator mechanism is the sum of kinetic energy of each component of the micro-manipulator, and total potential energy of the mechanism comes from the elastic deformation potential energy of the flexible spherical hinge and the gravitational potential energy of the micromanipulator.

When this flexible parallel micro-manipulator works, the sliders move along the vertical guide rail on the frame. So, its kinetic energy and potential energy can be expressed as

(14) $$T_{b}=\frac{1}{2}m_{b}\sum_{i=1}^{3}{\dot{b}}_{i}$$

(15) $$V_{b}=m_{b}g\sum_{i=1}^{3}b_{i}$$

where, ${\dot{b}}_{i}$ is the speed of the *i*-th slider, *m_b_* is the mass of the slider, and *g* is the acceleration of gravity.

The mobile platform of the mechanism has translational degrees of freedom along *x*, *y* and *z* directions, so their kinetic energy and potential energy of mobile platform can be expressed as

(16) $$T_{p}=\frac{1}{2}m_{p}({\dot{x}}^{2}+{\dot{y}}^{2}+{\dot{z}}^{2})$$

(17) $$V_{p}=m_{p}g(z+h)$$

where, *m_p_* is the mass of mobile platform.

Since the motion of the link rods is complicated and difficult to solve, the motion of the link rods is simplified in order to obtain its kinetic energy and potential energy. As shown in Figure 2b, each limb includes two link rods. It is assumed that the mass of the link rod *B_ij_P_ij_* is evenly distributed to its two ends. In this way, it can be considered that the motion of distributed masses at the upper and lower ends of the link rod are consistent with the mobile platform and the slider, respectively. Therefore, the total kinetic energy and potential energy of all the link rods can be respectively expressed as

(18) $$T_{c}=\frac{1}{2}m_{c}({\dot{x}}^{2}+{\dot{y}}^{2}+{\dot{z}}^{2})+\frac{1}{2}m_{c}\sum_{i=1}^{3}{\dot{b}}_{i}^{2}$$

(19) $$V_{c}=3m_{c}g(z+h)+m_{c}g\sum_{i=1}^{3}b_{i}$$

where, *m_c_* is the mass of one link rod.

This flexible parallel micro-manipulator mainly relies on elastic deformation of the flexible spherical hinge to produce movement [25]. Therefore, the elastic potential energy of the flexible spherical hinge should be considered in dynamic modeling. As shown in Figure 2b, there are four flexible spherical hinges in each limb, and deformations of these flexible spherical hinges are the same. Therefore, the total elastic potential energy expression of all the flexible spherical hinges can be written as

(20) $$V_{k}=\frac{1}{2}\sum_{i=1}^{3}4K_{b}\Delta \psi_{i}^{2}$$

with the notation of

$$K_{b}=diag(\begin{array}{ccc} k_{bm} & k_{bm} & 0 \end{array})$$

where ***K****_b_* represents the stiffness matrix of flexible spherical hinge. *k_bm_* is bending stiffness of the flexible spherical hinge, and it is determined by structural parameters of the flexible spherical hinge including minimum thickness *t_b_*, cutting radius *R* and maximum cutting angle *θ_m_* [26]. Since the stiffness of the flexible spherical hinge is not the focus of this paper, expression of *k_bm_* will not be expressed in detail here.

Lagrange’s function for the micro-manipulator can be generated as

(21) $$L=T_{b}+T_{p}+T_{c}-V_{b}-V_{p}-V_{c}-V_{k}$$

Lagrange’s equation of motion can be derived based on the generalized coordinates *q*, according to

(22) $$\frac{d}{dt}\left(\frac{dL}{d{\dot{q}}_{i}}\right)-\frac{dL}{dq_{i}}=F_{i}$$

where *q_i_* represents *i*-th generalized coordinate, *F_i_* is *i*-th actuation force.

The generated dynamic equations take on the following form

(23) $$M\ddot{q}+Kq+G=F$$

where ***M*** is the mass matrix, ***K*** is the stiffness matrix, ***G*** is the gravity force vector matrix, and ***F*** is the actual force matrix.

The natural frequency analysis of the micro-manipulator can not only avoid the resonance phenomenon of the platform when the platform is driven at high frequency, but also explore the relationship between the structural parameters and the natural frequency of the platform to guide the optimal design. According to Equation (23), the undamped free vibration dynamic equation of the micro-manipulator system can be expressed as

(24) $$M\ddot{q}+Kq=0$$

Respectively, where

$$\begin{array}{c} M=m_{p}E+m_{b}J^{T}J+m_{c}J^{T}J+m_{c}E\\ K=4\sum_{i=1}^{3}J_{\psi i}^{T}K_{b}J_{\psi i}^{T}+3(m_{b}+m_{c})g/hE_{h} \end{array}$$

Among them,

$$E_{h}=diag\left(\begin{array}{ccc} 1 & 1 & 0 \end{array}\right)$$

The condition of non-zero solutions for Equation (24) can be derived by

(25) $$\left|K-\omega_{0}^{2}M\right|=0$$

Natural frequencies of the micro-manipulator can be calculated by

(26) $$f=\frac{\omega_{0}}{2\pi }$$

## 5. Examples and Simulation Analysis

### 5.1. Numerical Examples and Modal Analysis

In order to verify the accuracy of the established dynamic model, numerical example analysis and finite element simulation analysis are carried out, respectively. The material of this micro-manipulator is chosen to be 65 Mn steel. Young’s modulus *E* and Poisson’s ratio *u* are 0.3 and 200 Gpa, respectively. And the density ρ is 7.85 g/cm^3^. Dimension parameters of the micro-manipulator are shown in Table 1.

According to the literature [26] and the dimension parameters of the flexible spherical hinge form Table 2, the bending stiffness of the flexible spherical hinge in this paper is 5.18 N/m. Then, natural frequencies of the micro-manipulator can be gained by employing Equation (26) using MATLAB software. Results of natural frequencies corresponding with three main directions of motion are 0 Hz (*z* direction), 56.16 Hz (*x* direction) and 56.16 Hz (*y* direction), respectively.

**Figure 4:**
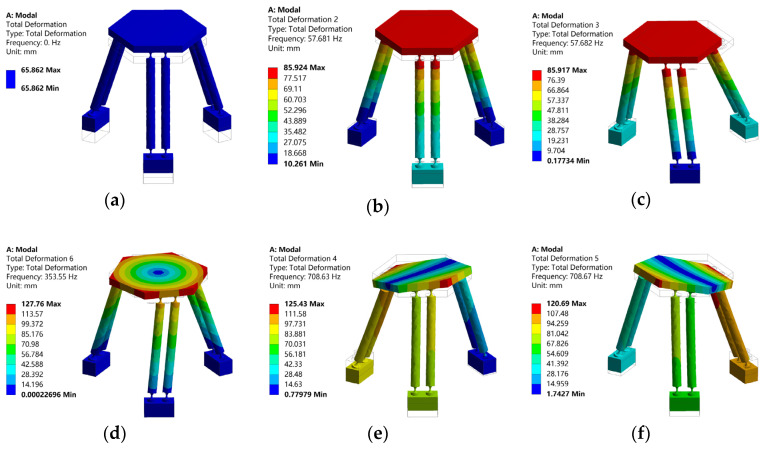
The first six modal shapes of the micro-manipulator. (**a**) Modal shape 1. (**b**) Modal shape 2. (**c**) Modal shape 3. (**d**) Modal shape 4. (**e**) Modal shape 5. (**f**) Modal shape 6.

For comparison purpose, a simulated modal analysis of the micro-manipulator with the same geometric and physical parameters (given in Table 1) was also performed by employing finite element ANSYS software. Results of the first six modal shapes of the micro-manipulator are shown in Figure 4 and Table 2. It can be seen that the frequencies of 4th, 5th and 6th order are much higher than those of 1st, 2nd and 3rd order. Therefore, only the first three frequencies are discussed in this paper.

From the simulation results, it can be seen that natural frequencies of the mechanism in *x* and *y* directions are almost equal, which is consistent with the conclusion of numerical calculation results. As shown in Table 3, the maximum error between numerical calculation results and finite element simulation results is 2.71%. Simulation results of second and third natural frequency are slightly larger than numerical results. Main reason for this phenomenon is that the link rods are regarded as rigid rods in theoretical dynamic modeling, and the strain energies generated by the elastic deformations of the link rods are ignored. Since the motion of the whole mechanism along the *z* direction is a rigid body motion without elastic deformation, natural frequency of the mechanism in the *z* direction is 0 Hz, which is also consistent with the numerical calculation results. Overall, the consistency of numerical calculation results and simulation results verify the correctness of the theoretical dynamic model of the 3-PSS flexible parallel micro-manipulator.

### 5.2. The Influence of Basic Structural Parameters Changes on Natural Frequencies

For structural optimization, the influence of basic structural parameters changes on the natural frequencies of the system is further analyzed here. According to the results of the above analysis, natural frequencies of *x* and *y* directions are equal, and the natural frequency of the *z* direction is 0 Hz. Thus, only the *x* direction (or *y* direction) needs to be considered when analyzing the influence of the basic parameters of the micro-manipulator on the natural frequencies. It can be seen from Equation (26) that the main parameters affecting the natural frequency of the system include the stiffness *k_bm_* of the flexible spherical hinge, the masses of main components (sliders, link rods and mobile platform), the difference in radius *E_r_* between the static platform and mobile platform and the length *l* of the link rod.

#### 5.2.1. Influence of the Stiffness k_bm_ of Flexible Spherical Hinge on the Natural Frequency

**Figure 5:**
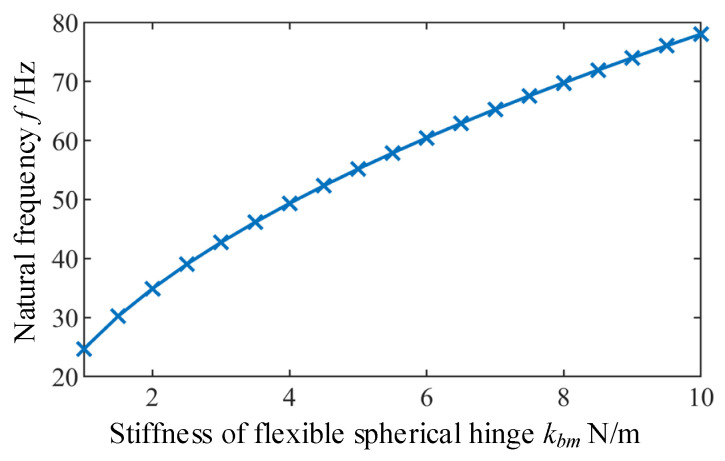
The influence the stiffness of flexible spherical hinge on the natural frequency of the micro-manipulator.

Since bending stiffness of the flexible spherical hinge in this example is 5.18 N/m, the stiffness change range is selected as 1–10 N/m. By increasing the bending stiffness of the flexible spherical hinge from 1 N/m to 10 N/m, corresponding changes of natural frequencies can be obtained according to Equation (26), as shown in Figure 5. It can be seen that natural frequency of the mechanism increases with the increase of the stiffness of the flexible spherical hinge *k_bm_*. However, it should be noted that increase of the stiffness of the flexible spherical hinge *k_bm_* will reduce the working space of the mechanism [27,28].

#### 5.2.2. Influence of the Mass of Main Component on the Natural Frequency

**Figure 6:**
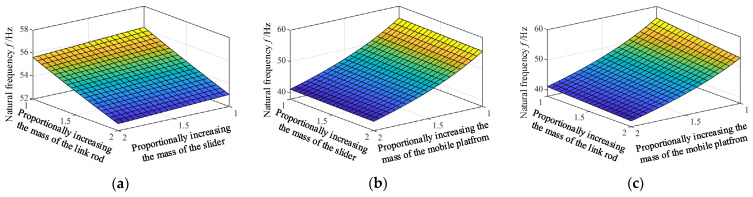
The influence of the variation of the mass proportional of different components on the natural frequency. (**a**) Proportionally increasing the mass of the link rod and the slider. (**b**) Proportionally increasing the mass of the slider and mobile platform. (**c**) Proportionally increasing the mass of link rod and mobile platform.

When the size structure of the component increases, the mass of the component will increase proportionally. So, when analyzing the influence of the masses changing on the natural frequency, it is assumed that their masses increase proportionally. And the impact of stiffness changes resulting from structural size changes is assumed to be ignored. The mass ratio of the link rod, the slider and the mobile platform are assumed to change from 1 to 2. Corresponding frequency changes can be obtained according to Equation (26), as is shown in Figure 6. It can be seen from Figure 6 that the increase in the mass of all components will decrease the natural frequency of the mechanism. In addition, compared with the slider and the link rod, the increase in the mass ratio of the mobile platform makes the natural frequency drop more sharply. This is because mass of original mobile platform is much larger than that of link rod and slider.

**Figure 7:**
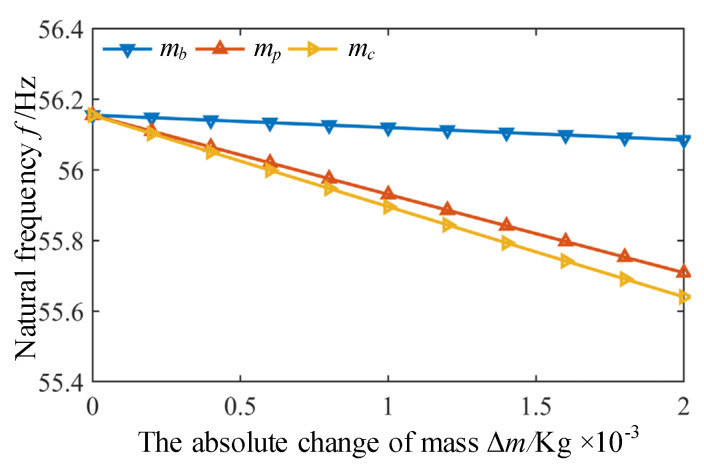
The influence of the absolute change of mass of different components on the natural frequency.

Considering that sometimes it is necessary to modify the designed structure to meet the requirements of specific dynamic characteristics, and the structural modification can only be carried out by changing local mass of original structure [29]. Therefore, influence of absolute change of mass of link rod, slider and mobile platform on the natural frequency is further analyzed. By adding extra mass Δ*m* (varying from 0 to 2 × 10^−3^ Kg) respectively to the link rod, the slider and the mobile platform, the corresponding changes of natural frequency can be obtained according to Equation (26), as shown in Figure 7. Obviously, when the same mass Δ*m* is added to different component, the link rod has the greatest impact on the natural frequency, followed by the mobile platform, and smallest the slider.

#### 5.2.3. The Influence of the Variations of the Length *l* of Link Rod and the Difference in Radius *E_r_* on the Natural Frequency

**Figure 8:**
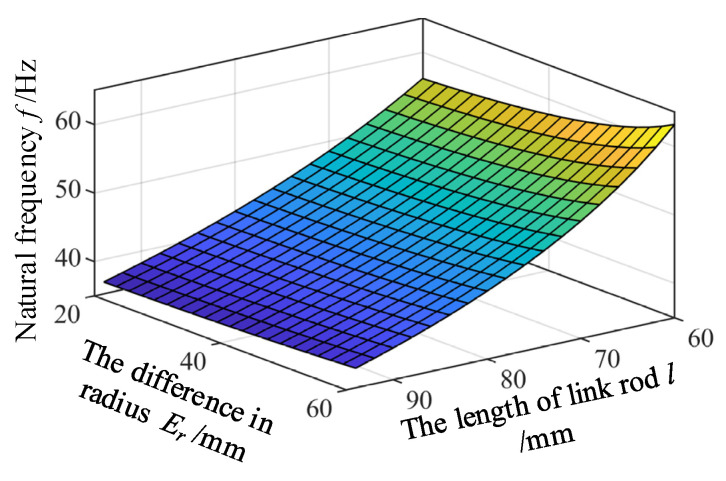
The influence of the variations of the length *l* of link rod and the difference in radius *E_r_* on the natural frequency.

When analyzing the influence of the difference in radius *E_r_* between the static platform and mobile platform on the natural frequencies, it is assumed that the radius *r_p_* of the mobile platform is kept unchanged. In addition, it is assumed that the mass *m_c_* of the link rods remains unchanged when the length *l* of the link rods changes. Variation ranges of the difference in radius *E_r_* and the length of the link rod are set to be 20–60 mm and 65–100 mm, respectively. Corresponding variation of the natural frequency can be obtained according to Equation (26), as shown in Figure 8. It can be seen from Figure 8 that natural frequencies of the mechanism increase as the difference in radius *E_r_* becomes larger, while decrease as the length *l* of the link rods increases.

**Figure 9:**
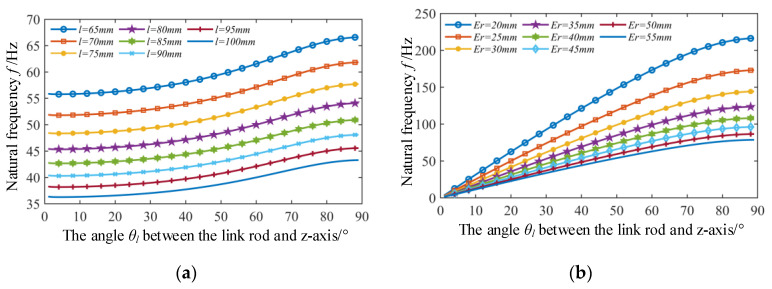
The change of natural frequency with variation of angle *θ_l_*_._ (**a**) Under the condition of different link rod length *l*. (**b**) Under the condition of different radius difference *E_r_*.

**Figure 10:**
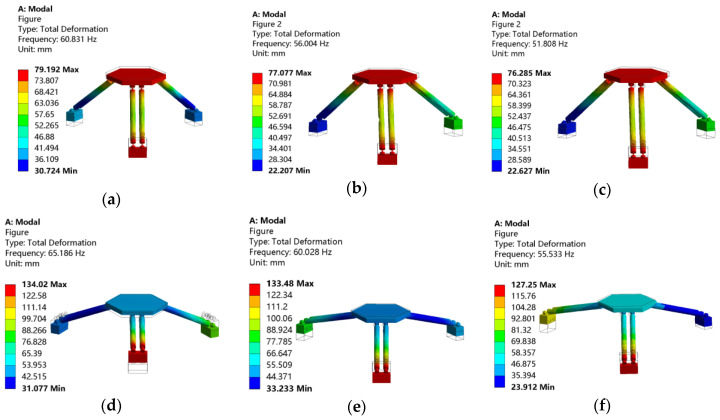
Modal analysis of models with different length *l* and angle *θ_l_*. (**a**) *l* = 65 mm, *θ_l_* =45°. (**b**) *l* = 70 mm, *θ_l_* =45°. (**c**) *l* = 75 mm, *θ_l_* =45°. (**d**) *l* = 65 mm, *θ_l_* =70°. (**e**) *l* = 70 mm, *θ_l_* =70°. (**f**) *l* = 75 mm, *θ_l_* = 70°.

Since the radius difference *E_r_* and the length *l* of the link rod determine the angle *θ_l_* between the link rod and the *z*-axis of the reference coordinate system, the influence of change of the angle *θ_l_* on the natural frequency is further analyzed. According to Figure 8, the influence of variation of the angle *θ_l_* on the natural frequency under different link rod lengths and different radius difference can be obtained, as shown in Figure 9a,b, respectively. Both Figure 9a,b show that the natural frequencies increase with the increase of the angle *θ_l_* under the condition of any given link rod length *l* or radius difference *E_r_*. Obviously, increasing the angle *θ_l_* is a good way for increasing the natural frequencies of the structure in structural design. However, it should be noted that increase of *θ_l_* will reduce the working space of the mechanism [28]. Furthermore, it can be seen in Figure 9a that under different link rod length conditions, the angle change has approximate effect on the change of the natural frequency. And Figure 9b shows that the smaller the radius difference *E_r_*, the greater the influence of the angle change on the natural frequency. To further verify the correctness of the above conclusions, modal analysis is carried out on the different simulated models with different structural parameters collected from Figure 9. For the sake of brevity, only six points are collected from Figure 9a. According to the structural parameters corresponding to the six points, six structural models can be established and modal analysis can then be carried out by ANSYS software. Since the frequencies of the first-order models of the modal analysis of different models are all 0 Hz, and the frequencies of the second-order and third-order models are approximately equal. For simplicity, the second-order mode shape is given here. The frequency and mode shape results are shown in Figure 10.

Picking out numerical calculation results corresponding to these six points from Figure 9, comparison can then be made, as shown in Table 4. It is demonstrated in Table 4 that the maximum error between numerical calculation results and finite element simulation results is only 3.64%, which further proves the correctness of the theoretical modeling established before. In addition, it can be seen from Table 4 that when the rod length is 65, 70 and 75 mm respectively, increment of the natural frequency resulting from changes of the angle *θ_l_* from 45° to 70° are relatively close, all about 5 Hz. This also verifies the correctness of the previous conclusions that “under different link rod length conditions, the angle change has approximate effect on the change of the natural frequency.”

## 6. Conclusions

The Lagrangian method is used to establish the dynamic model of the 3-PSS flexible parallel micro-manipulator, the expression of natural frequencies is further obtained. The results of numerical calculation and modal simulation analysis are obtained through MATLAB software and ANSYS software, respectively. Comparing the numerical calculation results with the results of finite element analysis, the maximum error of their results is 2.71%, which verifies the correctness of the dynamic model. Then, influence of the basic parameters of the mechanism on the natural frequencies is analyzed. Analysis results show that the natural frequencies increase with the increase in the bending stiffness *k_bm_* of the flexible spherical hinge and the difference in radius *E_r_* between the static platform and the mobile platform, while decrease with the increase of the length *l* of link rod and the mass of the main components of mechanism increase. In addition, it can be further inferred from the obtained results that the natural frequencies increase with the increase of the angle *θ_l_* between the link rod and the *z*-axis of the reference coordinate system.

Considering that increase of the stiffness of the flexible spherical hinge *k_bm_* and the angle *θ_l_* will reduce the scope of the working space, it is recommended in designing the structure to choose a larger *θ_l_* as much as possible under the premise of satisfying the working space. Therefore, during structural design, it is recommended to choose a relatively larger stiffness *k_bm_* and angle *θ_l_* under the premise of ensuring working space requirements. Research of this paper can provide a theoretical basis for the structural optimization design of the 3-PSS flexible parallel micro-manipulation.

## Figures and Tables

**Table 1:** Dimension parameters of the micro-manipulator.

Parameter	*r_p_*/mm	*r_a_*/mm	*l*/mm	*t_b_*/mm	*R*/mm	*θ_m_*/°
Numerical value	45	25	65	1	2.5	60

**Table 2:** The first six natural frequencies of the micro-manipulator.

No	1	2	3	4	5	6
Natural Frequencies *f*/Hz	0	57.681	57.682	353.35	708.63	708.67

**Table 3:** Comparison of theoretical and simulation results of the natural frequencies.

No	Natural Frequencies *f/*Hz		Relative Error/%
	Theoretical Value	Simulation Value	
1	0	0	0
2	56.16	57.681	2.71
3	56.16	57.682	2.71

**Table 4:** Comparison of numerical calculation results and finite element simulation of six models with different parameters.

	Parameters	*l =* 65 mm		*l =* 70 mm		*l =* 75 mm	
*f*/Hz		*θ_l_ =* 45*°*	*θ_l_ =* 70*°*	*θ_l_ =* 45*°*	*θ_l_ =* 70*°*	*θ_l_ =* 45*°*	*θ_l_ =* 70*°*
Numerical results/Hz		58.74	63.81 z	54.55	59.27	50.91	55.3
Simulation results/Hz		60.88	65.186	56.004	60.028	51.808	55.533
Relative error/%		3.64	2.15	2.66	1.27	1.76	0.42
